# The Landscape of Featured Metabolism-Related Genes and Imbalanced Immune Cell Subsets in Sepsis

**DOI:** 10.3389/fgene.2022.821275

**Published:** 2022-02-21

**Authors:** Han She, Lei Tan, Yuanqun Zhou, Yu Zhu, Chunhua Ma, Yue Wu, Yuanlin Du, Liangming Liu, Yi Hu, Qingxiang Mao, Tao Li

**Affiliations:** ^1^ Department of Anesthesiology, Daping Hospital, Army Medical University, Chongqing, China; ^2^ State Key Laboratory of Trauma, Burns and Combined Injury, Second Department of Research Institute of Surgery, Daping Hospital, Army Medical University, Chongqing, China

**Keywords:** sepsis, biomarkers, bioinformatics, immune cell infiltration, metabolomics

## Abstract

Sepsis is a heterogeneous disease state triggered by an uncontrolled inflammatory host response with high mortality and morbidity in severely ill patients. Unfortunately, the treatment effectiveness varies among sepsis patients and the underlying mechanisms have yet to be elucidated. The present aim is to explore featured metabolism-related genes that may become the biomarkers in patients with sepsis. In this study, differentially expressed genes (DEGs) between sepsis and non-sepsis in whole blood samples were identified using two previously published datasets (GSE95233 and GSE54514). A total of 66 common DEGs were determined, namely, 52 upregulated and 14 downregulated DEGs. The Gene Set Enrichment Analysis (GSEA) results indicated that these DEGs participated in several metabolic processes including carbohydrate derivative, lipid, organic acid synthesis oxidation reduction, and small-molecule biosynthesis in patients with sepsis. Subsequently, a total of 8 hub genes were screened in the module with the highest score from the Cytoscape plugin cytoHubba. Further study showed that these hub DEGs may be robust markers for sepsis with high area under receiver operating characteristic curve (AUROC). The diagnostic values of these hub genes were further validated in myocardial tissues of septic rats and normal controls by untargeted metabolomics analysis using liquid chromatography-mass spectrometry (LC-MS). Immune cell infiltration analysis revealed that different infiltration patterns were mainly characterized by B cells, T cells, NK cells, monocytes, macrophages, dendritics, eosinophils, and neutrophils between sepsis patients and normal controls. This study indicates that metabolic hub genes may be hopeful biomarkers for prognosis prediction and precise treatment in sepsis patients.

## Introduction

Sepsis, a life-threatening organ dysfunction induced by an exaggerated, uncontrollable immune response to an infection, is one of the major public health concerns with high hospitalization and mortality rate (Singer et al., 2016; Scott, 2017). According to a systematic review extrapolated from published population estimates to the global population, approximately 30 million sepsis episodes and 6 million sepsis-related mortality occur per year (Fleischmann et al., 2016). In mainland China, sepsis affects one-fifth of intensive care unit (ICU) admissions with a 90-day mortality rate of 35.5% (Xie et al., 2020). Despite enormous international efforts and achievements in multi-disciplinary approaches, sepsis remains a major contributor to the growing global burden of morbidity and mortality (Rudd et al., 2018). Rapid early diagnosis and proper management are necessary to improve the outcomes in patients with sepsis (Rhodes et al., 20162017). Besides symptoms and signs for the early diagnosis of sepsis, available biomarkers are important for the following treatment of sepsis. Some biological markers have been established such as lactate, C-reactive protein (CRP), procalcitonin (PCT), B-type natriuretic peptide (BNP), and soluble RAGE (sRAGE) in sepsis, while lack of sensitivity and specificity limit their clinical application (Oberhoffer et al., 1999; Phua et al., 2008; Hamasaki et al., 2014; Débora Maria da Gomes Cunha and Yoshio Hamasaki, 2019). It is of prime importance to identify more effective technologies and specific biomarkers for sepsis.

The early organ failure of sepsis typically occurs without grossly evident histological changes, while the metabolic and immune responses to sepsis occur early and have associations with organ failure (Michie, 1996; Singer, 2014; Lewis et al., 2016). Ignorance of changes in cell metabolic and immune functions may lead to the failure of sepsis treatment (Koutroulis et al., 2019). Therefore, metabolic and immune biomarkers are needed to predict prognosis of sepsis.

An increasing number of studies on diseases are focusing on the role of metabolic changes that occur during the development of diseases and can also lead to subsequent pathophysiological changes of diseases. The metabolome, the underlying biochemical layer of the host’s genome, transcriptome, and proteome, can reflect all the external and internal cellular activities modulated throughout all other omic layers (Jacob et al., 2019; Djande et al., 2020). Given that the metabolome is closely associated with the phenotype, metabolism-related genes provide new opportunities for personalized diagnosis, monitoring, and treatment towards specific pathophysiological mechanisms related to metabolism in patients (Zhang et al., 2015; Itenov et al., 2018). Among the mechanisms of sepsis and the development of organ dysfunction, various molecules and miRNAs have been studied, such as IL-17A, TLR4, C5a, MIF, miR-132, miR-146, miR-150, and miR-155 (Pop-Began et al., 2014). Metabolic reprogramming has also been observed early in sepsis and is proposed to be adaptive to limit additional injury, maintain energy balance, preserve cellular composition, and prevent DNA damage (Singer et al., 2004). The use of metabolomics in sepsis patients may provide new approaches to explore sepsis markers and enable precision medicine.

Over the last few decades, the diagnostic criteria for sepsis have being updated periodically, but the primary pathophysiology remains constant—the presence of inflammation during disease (Nedeva et al., 2019). However, patients with sepsis may have distinct inflammatory responses and unique profile of immune activation against the pathogen (Pinheiro da Silva and César Machado, 2015). Therefore, the desirable way to decipher the heterogeneity and advance the treatment of sepsis may be identifying companion biomarkers for better assessing immune status and stratifying patients (Venet and Monneret, 2018).

The complex inflammatory responses and metabolism-related genes during sepsis have not been fully elucidated. Herein, we integrated two datasets to identify differentially expressed metabolism-related genes using a support vector machine (SVM) and further explored the value of these genes in the diagnosis of sepsis. The immune cell infiltration analysis between sepsis patients and normal controls were further performed by CIBERSORT. Metabolic mechanisms underlying the inflammatory responses during sepsis were also investigated by using LC-MS to identify potential novel therapeutic targets and pathways for patients with sepsis.

## Materials and Methods

### Ethics Statement

The study protocol was approved by the Research Council and Animal Care and Use Committee of the Research Institute of Surgery, Daping Hospital, Army Medical University (No. DHEC-2012-069). All the animal experiments were performed in accordance with the principles of the *Guide for the Care and Use of Laboratory Animals* set forth by the United States National Institutes of Health (NIH Publications, 8th edition, 2011).

### Animal Management

Adult female and male Sprague–Dawley rats (*n* = 22) weighing 200–220 g were purchased from the Experimental Animal Center of the Research Institute of Surgery, Daping Hospital, Army Medical University. All animals were bred in the animal facility under filtered positive-pressure ventilation on a 12:12-h dark/light cycle. The temperature and relative humidity of the breeding room were maintained constant at 23–25°C and 55%–65%, respectively. The rats were allowed to acclimatize for 3 days before use and they were solid-food restricted with water *ad libitum* for 8 h prior to the initiation of surgery.

### Sepsis Model Establishment and Sample Preparation

The rats were randomly divided into two groups: sepsis and control group. They were anesthetized for the surgical procedure with sodium pentobarbital (45 mg/kg, i.p.), which is a short-acting anesthetic agent with limited impact on cardiovascular functions for anesthesia. Cecal ligation and puncture were performed to reproduce a previously described sepsis model (Zhu et al., 2016). Briefly, the cecum was carefully exposed, ligated after a midline laparotomy, and punctured 0.7 cm from the distance with a 1.5-mm-diameter triangular needle, allowing fecal matter to flow into the abdominal cavity freely. After closure of the abdomen, the rats were returned to the cages with *ad libitum* access to food and water. Myocardial tissues were harvested at 12 h after CLP and samples were stored at −80°C until analysis.

### Microarray Data

To identify differentially expressed genes (DEGs), the microarray data for sepsis were downloaded from the Gene Expression Omnibus (GEO) database (https://www.ncbi.nlm.nih.gov/geo/) under accession numbers GSE95233 (Tabone et al., 2018) and GSE54514 (Parnell et al., 2013). To explore a prognostic gene expression signature for sepsis, dataset GSE95233 was used as the training dataset and then applied to verify the performance of the SVM classifier. Background correction and quantile normalization were performed using linear models for the microarray data (LIMMA) software package. All these data were freely available online.

### Screening of Differentially Expressed Metabolism-Related Genes

The metabolism-related genes were obtained from the Molecular Signatures database (MSigDB) v7.4 (http://software.broadinstitute.org/gsea/msigdb) by searching using the term “metabolism”. The C2sub-collection (c2. cp.kegg.v7.4. symbols.gmt) was selected as the reference gene set. Next, we extracted the metabolism-related gene expression matrix from the GSE95233 and GSE54514 datasets using the merge function in R. The analysis of differentially expressed metabolism-related genes between sepsis patients and normal controls was conducted using the LIMMA package implemented in the R statistical package. The threshold for the identification of DEGs was set at a *p* value of <0.05. Then, these DEGs were divided into upregulated and downregulated DEGs in each dataset.

### Functional Enrichment Analysis of DEGs by DAVID, FunRich, and GSEA

In order to analyze the functions of DEGs, Gene Ontology (GO) analysis was performed using the DAVID online database. The biological functions of DEGs were validated by the FunRich platform. The cellular components, molecular function, biological process, and biological pathway of these DEGs were then analyzed by using FunRich platform. *p* < 0.05 was considered statistically significant. At the same time, the gene set enrichment analysis (GSEA) was performed using the GSEA 4.1.0 software. The MSigDB of c2 (c2. cp.kegg.v7.4. symbols.gmt) was used as the reference gene set in GSEA. The number of permutations was set to 1,000 and the phenotype labels were high and low expression. FDR <0.25 and *p* < 0.05 were set as the cutoff criteria to confirm significant gene sets.

### Analysis of Hub Genes by String and GeneMANIA

The STRING database (https://string-db.org/) focuses on collecting, scoring, and integrating known and predicted protein–protein interaction (PPI) information. We first conducted a PPI network analysis of differentially expressed hub genes to explore the potential interactions *via* STRING. Next, the most important hub genes were screened using the Cytoscape software plugin cytoHubba. GeneMANIA (http://www.genemania.org) is an online analysis tool providing information to predict the function of genes and gene sets, protein and genetic interactions, pathways, co-expression, co-localization, and protein domain similarity of submitted gene lists. In the present study, GeneMANIA was applied to construct a gene–gene interaction network for hub genes.

### Real-Time Quantitative RT-PCR (qRT-PCR)

RNA was extracted from rat myocardial tissues using TRIzol reagent according to the manufacturer’s instructions (Cat#15596018, Thermo). cDNA was synthesized using an RT reagent Kit with gDNA Eraser (Perfect Real Time) for real-time quantitative qRT-PCR (Cat#RR047A, Takara). SYBR Premix Ex Taq II (TliRNaseH Plus) (Cat#RR820B, Takara) was applied to analyze mRNA expression of hub genes. The relative RNA expression levels were calculated with the efficiency corrected 2^–ΔΔCT^ method using β-actin as an internal control. Gene-specific primer pairs used in this experiment are listed in Table1.

**TABLE 1:** PCR primer sequences of hub genes.

Genes	Forward primers	Reverse primers
Pole4	CTT​GGT​GAA​GGC​AGA​CCC​TG	AGC​ACA​GCA​GTA​GGC​ATC​TT
Pold4	ATG​GGC​CTT​GCA​CAG​GTA​TC	AGT​GGG​TAG​AGA​TGC​CAG​AGG
Polr21	GAT​CAT​CCC​AGT​TCG​CTG​CT	CAT​CCC​CCT​CGG​TGT​ACT​CT
Polr2j	ACG​CTT​GCT​TGT​TCA​CCA​TC	GGG​GTG​AGG​GAC​TTT​GTA​GC
Nme6	CTG​CCG​GAG​GTT​TTA​CCG​AG	AGG​ATG​TAG​GCT​CGG​ATT​GG
Nme1	TCT​CGG​GGA​ACC​TAC​ATC​CTG	GGC​TGT​TCA​GCT​GGG​ATC​AT
Entpd1	TCT​CGG​GGA​ACC​TAC​ATC​CTG	GGC​TGT​TCA​GCT​GGG​ATC​AT
Adcy3	ATG​TTG​CAC​GCC​ATT​TCC​TG	GGC​AAG​GAG​GCA​AAC​ATG​AC

### Hierarchical Clustering Analysis for Hub Genes and Support Vector Machine Classifier Construction

Hierarchical clustering of hub genes was applied to explore the differences in expression patterns between sepsis patients and healthy controls in the training dataset GSE95233. The average linkage method was used to conduct hierarchical clustering and Pearson correlation method was used to set the clustering distance in ClustVis (http://biit.cs.ut.ee/clustvis/) (Metsalu and Vilo, 2015). SVM was used to construct a classification model on the basis of the optimal feature subset with a 10-fold cross-validation method for identifying sepsis patients from healthy controls. A machine learning method that combined SVM with recursive feature elimination (RFE) was applied to construct a classifier verifying the hub genes that can discriminate sepsis patients from normal controls. The receiver operating characteristic (ROC) curve was constructed, and the area under the ROC curve (AUC) was calculated in MedCalc (version 14.10.20, http://www.medcalc.org/).

### Analysis of Immune Cell Infiltration

Based on the gene expression matrix, CIBERSORT software was used to characterize the relative proportion and corresponding *p*-value of 22 immune cells in each sample in the training dataset GSE95233. The statistical significance of the deconvolution consequences of all cell subsets was reflected by the CIBERSORT *p*-value. The number of permutations was set to 1,000, and a threshold *p*-value of <0.05 was the criterion for successful computation of the samples. Then, heatmaps were constructed to visualize differentially infiltrated immune cells between sepsis and healthy control. Pearson’s correlation analysis in R software was used to investigate correlation matrix in all pairs of immune cells and the association of the hub genes with infiltrating immune cells, respectively.

### Cytoscape

Cytoscape 3.7.2 (https://js.cytoscape.org/) was used to the visualize biomedical networks composed of metabolic and gene interactions. MetScape 3 (http://MetScape.ncibi.org) is a Cytoscape plug-in for building and analyzing networks of genes and compounds, identifying enriched pathways, and visualizing changes in metabolite data. The data obtained from metabolites and the hub genes identified between healthy control and sepsis patients were imported into MetScape to detect the metabolomics network regulated by the hub genes.

### Metascape

Metascape (http://metascape.org/gp/index.html) was employed for integrated functional enrichment, gene annotation, and interactive group analysis. The hub genes were inputted into Metascape for producing enriched pathways of hub genes.

### Metabolomics Profiling

Metabolomics profiling was performed in tissues of septic rats and sham control by using a UHPLC system (Vanquish, Thermo Fisher Scientific) with a UPLC BEH Amide column (2.1 mm × 100 mm, 1.7 μm) coupled to a Q Exactive HFX mass spectrometer (Orbitrap MS, Thermo, United States). The QE HFX mass spectrometer was used to acquire MS/MS spectra on information-dependent acquisition (IDA) mode in the control of the acquisition software (Xcalibur, Thermo, United States). The resulting three-dimensional data involving the peak number, sample name, and normalized peak area were used as the input for performing principal component analysis (PCA) in the SIMCA14 software package (Umetrics, Umea, Sweden). The annotated differently expressed metabolites were illustrated as a Volcano plot. Chord diagram was applied for biomarker metabolites depicting distributions and links between potential metabolites. The expression levels of the significantly changed metabolites and metabolic pathway analysis between septic rats and sham group were analyzed by a heatmap and a bubble plot generated by TBtools (Chen et al., 2020), respectively.

### Statistical Analysis

All statistical analyses were performed in SPSS 17.0 (IBM, United States). The differentially expressed metabolism-related genes were identified using the Limma package with *p* < 0.05 as the criteria. Hierarchical clustering analysis was used for the identified featured genes using the heatmap package in R. The SVM classifier was constructed using the e1071 R package with 10-fold cross-validation. ROC analysis was performed and the AUC was calculated to evaluate the predictive performance of the classifier. The correlation of co-expression was adjusted by Pearson’s correlation, and the strength of the correlation was determined according to the following absolute value criteria: *r* = 0.00–0.19 (very weak), *r* = 0.20–0.39 (weak), *r* = 0.40–0.59 (moderate), *r* = 0.60–0.79 (strong), and *r* = 0.80–1.0 (very strong). *p* < 0.05 was considered to indicate a statistically significant correlation. All statistical analyses were performed using the R software (version 4.0.5, http://www.r-project.org).

## Results

### Identification of Differentially Expressed Metabolism-Related Genes

**FIGURE 1:**
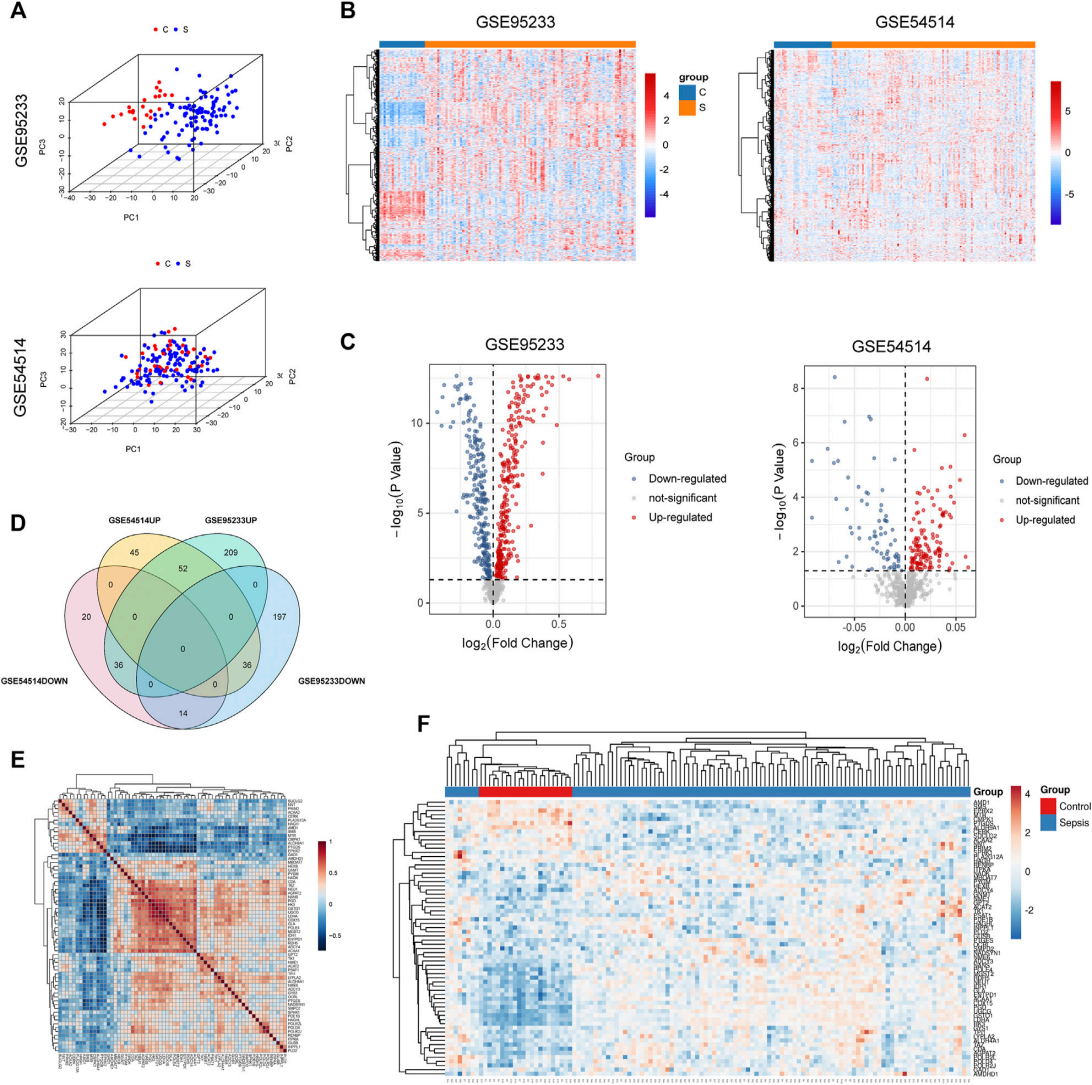
Metabolism-related genes are among DEGs found in sepsis patient versus healthy control whole blood. **(A)** Principal components analysis (PCA) score plot of GSE95233 and GSE54514. Red represents control patients, and blue represents sepsis patients. **(B)** Heatmap of GSE95233 and GSE54514. **(C)** Volcano plot of GSE95233 and GSE54514. Gray dots indicate downregulated DEGs while red dots indicate upregulated DEGs. Statistically significant DEGs were identified as those with Student’s t-test *p*-values < 0.05. **(D)** Venn diagram of DEGs identified from the two GEO datasets (-UP: upregulated DEGs, -DOWN: downregulated DEGs). **(E)** Co-expression network of the differentially expressed metabolism-related genes identified from GSE95233. Red depicts high gene expression and blue depicts low gene expression. **(F)** Heatmap of the 66 DEGs between patients with sepsis and healthy controls in the training cohort GSE95233.

The flow chart of this study is shown in Supplementary Figure S1. To identify sepsis-associated genes, we first analyzed genes differentially expressed in whole blood samples between sepsis and non-sepsis patients from the GEO database. As shown in the results of the PCA, gene expression patterns were significantly different between sepsis patients and healthy people (Figure 1A). Compared with non-sepsis samples, a total of 544 DEGs were identified in GSE95233, 297 of which were upregulated and 247 were downregulated (Figures 1B,C). A total of 203 DEGs were observed in GSE54514, 133 upregulated and 70 downregulated (Figures 1B,C). Intersection analysis of these DEGs showed 66 genes, which were simultaneously differentially expressed in the two datasets, including 52 upregulated and 14 downregulated DEGs (Figure 1D). To explore the co-expression network of these differentially expressed metabolism-related genes, Pearson’s correlation coefficient was used to evaluate the correlation between a given pair of DEGs in the training cohort GSE95233 (Figure 1E). A heatmap of the 66 DEGs was constructed to compare sepsis patients and healthy controls in the training cohort GSE95233 (Figure 1F).

### Functional Enrichment Analysis of DEGs

**FIGURE 2:**
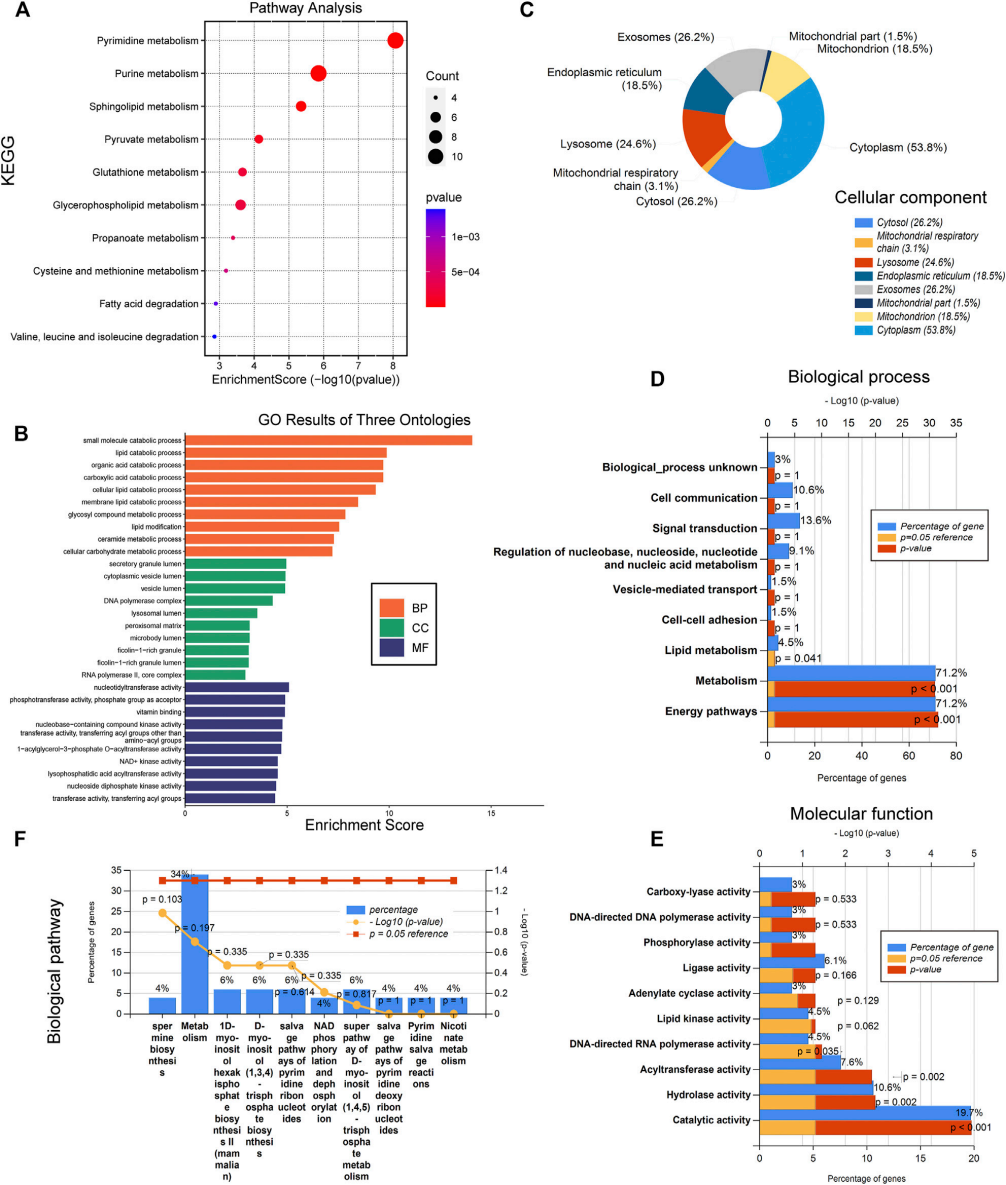
Metabolism-related pathways are involved in the pathophysiology of sepsis. **(A)** Kyoto Encyclopedia of Genes and Genomes (KEGG). **(B)** Gene ontology (GO) results of biological process (BP), cellular component (CC), and molecular function (MF). **(C)** Cellular component (CC). **(D)** Molecular function (MF). **(E)** Biological process (BP). **(F)** Biological pathway.

In order to elucidate the underlying functions of these differentially expressed metabolism-related genes in sepsis, enrichment analyses were performed using the DAVID 6.8 enrichment analysis. GO annotation analysis showed that these DEGs were involved in metabolic processes such as small-molecule catabolic process, lipid catabolic process, and so on (Figure 2B). Cellular component analysis showed that the DEGs were mainly related to secretory granule lumen, cytoplasmic vesicle lumen, lysosomal lumen, and so on (Figure 2B). Further molecular function analysis found that the common DEGs were engaged in metabolism-related nucleotidyltransferase activity, phosphotransferase activity, vitamin binding, nucleobase-containing compound kinase activity, and NAD^+^ kinase activity (Figure 2B). KEGG analysis was further utilized to define the possible pathways linked to the functions of these DEGs. The metabolic pathways were remarkably enriched, including pyrimidine metabolism, purine metabolism, sphingolipid metabolism, glycerophospholipid metabolism, pyruvate metabolism, and glutathione metabolism (Figure 2A). The above enrichment results were validated by the FunRich software v.3.1.3 that showed that the mitochondrial respiratory chain, catalytic activity, hydrolase activity, metabolism, and energy pathways were significantly enriched among DEGs (Figures 2C–F). Gene set enrichment analysis (GSEA) was then performed to further validate the pathways that were differentially enriched among DEGs. The significantly enriched signaling pathways were presented based on their normalized enrichment score (NES). As expected, “carbohydrate derivative metabolism”, “cellular lipid metabolism”, “lipid metabolism”, “organic acid metabolism”, “organonitrogen compound biosynthesis”, “organophosphate metabolism”, “oxidation reduction”, and “small molecule biosynthesis” were differentially enriched in the sepsis group compared to those in healthy controls (Supplementary Figure S2). These results indicated that metabolism-related pathways may be involved in the pathophysiology of sepsis.

### The Metabolomics Profiling in Heart Tissue of Septic Rats

**FIGURE 3:**
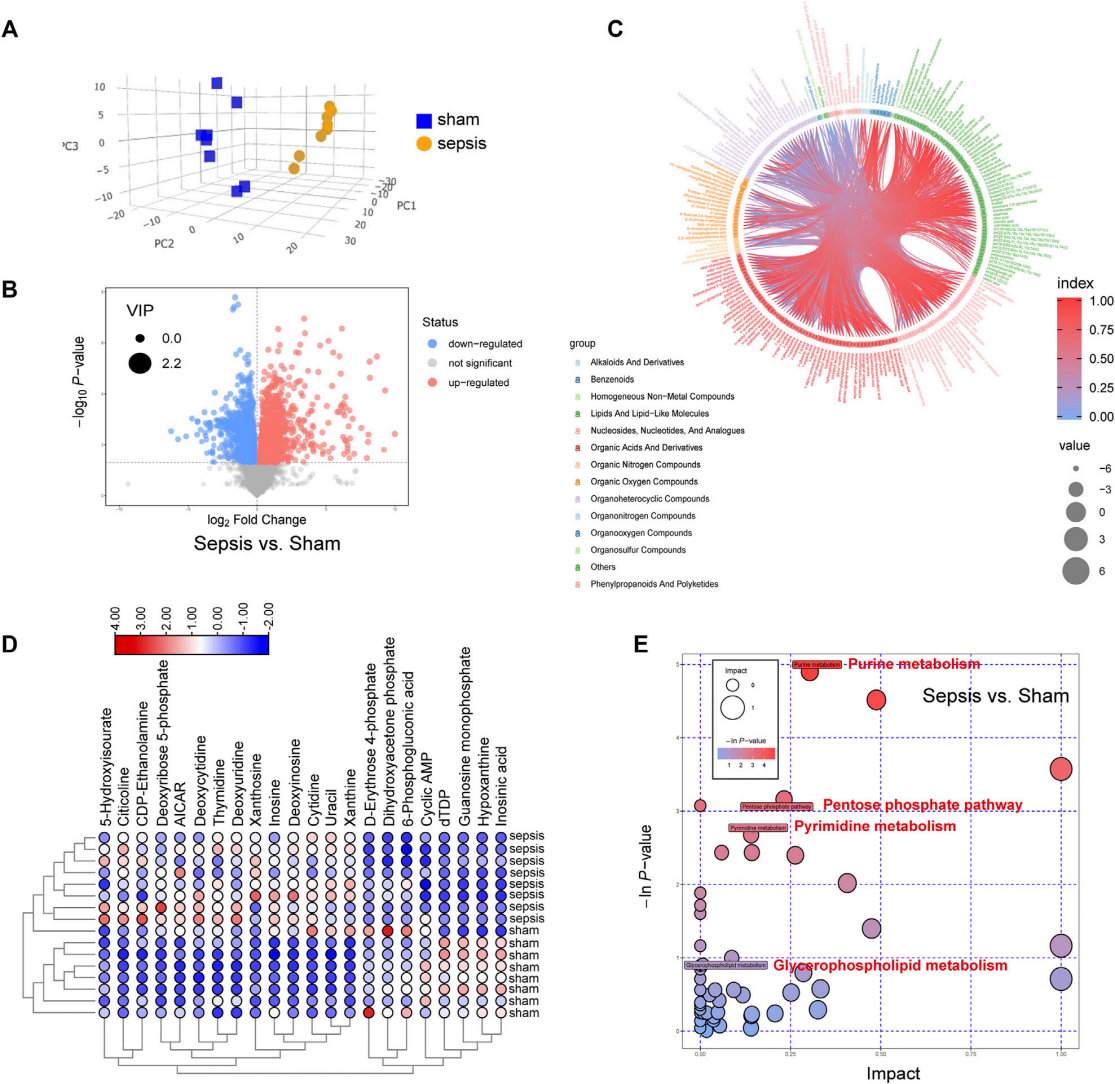
Metabolic changes were identified by metabolomics profiling in hearts of septic rats and sham controls. **(A)** Principal components analysis (PCA) score plot for metabolomics analysis in septic rats and normal control. Orange represents sepsis rats, and blue represents normal control. **(B)** Volcano plot, **(C)** circle diagram, and **(D)** heatmap analyzed by TBtools showing the significantly changed metabolites in septic rats and normal control. **(E)** Bubble plot of the metabolic pathway enrichment analysis identified in septic rats and normal control. The different color depths of circles represent the *p*-value of pathway enrichment analysis.

To verify the metabolomics change following sepsis, untargeted metabolomics analysis was performed in heart tissue following sepsis in rats by LC-MS. PCA score plot and volcano plot showed metabolite difference from discrimination analysis (Figures 3A,B). Then, a data-driven correlation analysis using the chord diagram of the group lasso-selected metabolites was performed to visualize the interplays between significant metabolites and their relevant metabolic pathways (Figure3C). Most interconnections arose between “lipids and lipid-like molecules”, “nucleosides, nucleotides and analogues”, “organic acids and derivatives”, and “organoheterocyclic compounds”. To compare the expression levels of the 22 metabolites identified to be significantly between septic rats and normal control, an interactive heatmap was constructed with Tbtools (Figure 3D). Moreover, pathway enrichment analysis found that the most significantly changed pathways were involved with glycerophospholipid metabolism, pyrimidine metabolism, purine metabolism, and pentose phosphate pathway (Figure 3E). The results validated that metabolism changes were a vital feature following sepsis.

### Protein–Protein Interaction Network and Hub Genes

**FIGURE 4:**
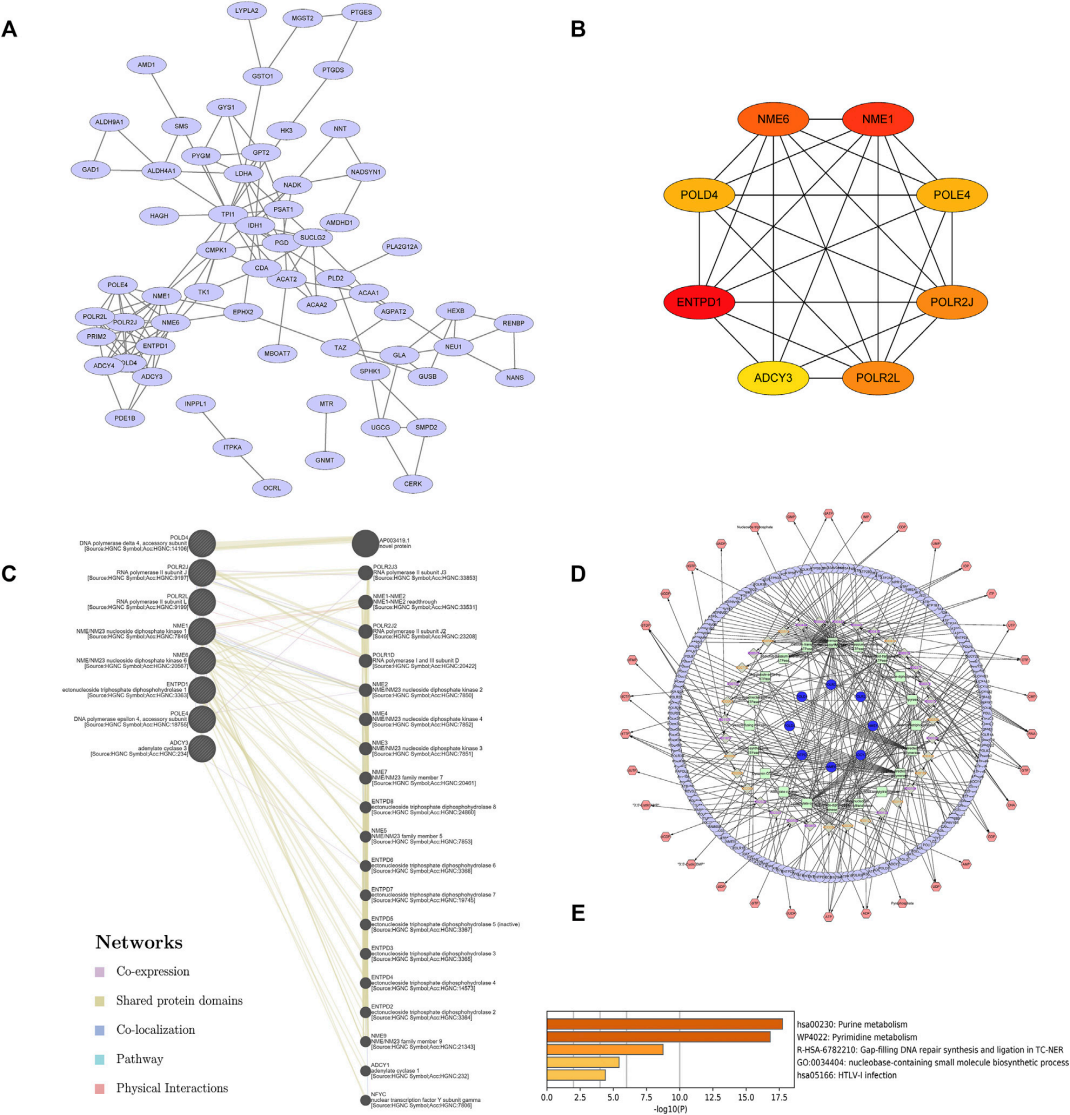
Hub genes were identified by the PPI network complex of metabolic DEGs. **(A)** Sixty-six DEGs with 66 nodes and 119 edges were displayed using STRING. **(B)** The 8 most important hub genes were screened using the Cytoscape software plugin cytoHubba. The PPI network data from STRING was further analyzed by Cytoscape and hub genes identification was performed by cytoHubba. **(C)** Gene–gene interaction networks and functions of 8 hub genes in GeneMANIA. **(D)**The landscape of metabolic network of hub genes. **(E)** Enriched pathways of hub genes in Metascape.

To further explore the potential interactions among these differentially expressed metabolism-related genes, a PPI network analysis was conducted with the STRING database. The 66 nodes and 119 edges in a PPI network were built among the 66 common DEGs (Figure 4A). The visual analysis and hub gene screening were presented by the Cytoscape software in combination with STRING results. The hub genes included *NME/NM23 Nucleoside Diphosphate kinase 1 (NME1)*, *NME6, DNA polymerase Delta4 Accessory Subunit (POLD4)*, *DNA polymerase Epsilon 4 Accessory Subunit (POLE4)*, *RNA polymerase II Subunit J (POLR2J)*, *RNA polymerase II, I And III Subunit L (POLR2L)*, *Adenylate cyclase 3 (ADCY3)*, and *Ectonucleoside Triphosphate diphosphohydrolase 1 (ENTPD1)* (Figure 4B). To further investigate their networks and functions, GeneMANIA was applied to construct their gene networks. A total of 20 nodes representing genes and 180 links were associated with the above 8 hub genes in co-expression, shared protein domains, co-localization, pathway, and physical interactions. The top 10 genes related to the hub genes were *AP003419.1, POLR2J3, NME1-NME2, POLR2J2, POLR1D, NME2, NME3, NME4, NME7*, and *ENTPD8*. Further functional analysis revealed that these hub genes were significantly correlated with nucleoside-diphosphatase activity (FDR = 5.37E-17), followed by nucleoside phosphate catabolic process (FDR = 1.75E-10), nucleoside diphosphate metabolic process (FDR = 4.07E-10), nucleobase-containing small-molecule biosynthetic process (FDR = 1.11E-9), organophosphate catabolic process (FDR = 1.01e-8), nucleotidyltransferase activity (FDR = 3.53E-7), and 5′-3′ RNA polymerase activity (Figure 4C). These results suggest that these hub genes played important metabolic roles in sepsis.

To detect the metabolomics network regulated by the hub genes, the network visualization of the compound, gene, enzyme, and reaction was conducted using Cytoscape and MetScape. Each node represents a metabolite and the edge represents the correlation coefficient between nodes (Figure 4D). The correlation-based metabolic network showed that the featured hub genes in sepsis played vital roles in various metabolism-related processes. The metabolic pathway analysis of hub genes was performed by Metascape (Figure 4E). A total of 5 metabolic pathways (*p* value <0.01) were related to hub genes in sepsis patients, namely, purine metabolism, pyrimidine metabolism, GAP-filling DNA repair synthesis and ligation in TC-NER, nucleobase-containing small-molecule biosynthetic process, and HTLV-I infection. The results indicated that there were 8 metabolic hub genes that might be predictors for sepsis.

### Diagnostic Value of Eight Featured Hub Genes in Sepsis

**FIGURE 5:**
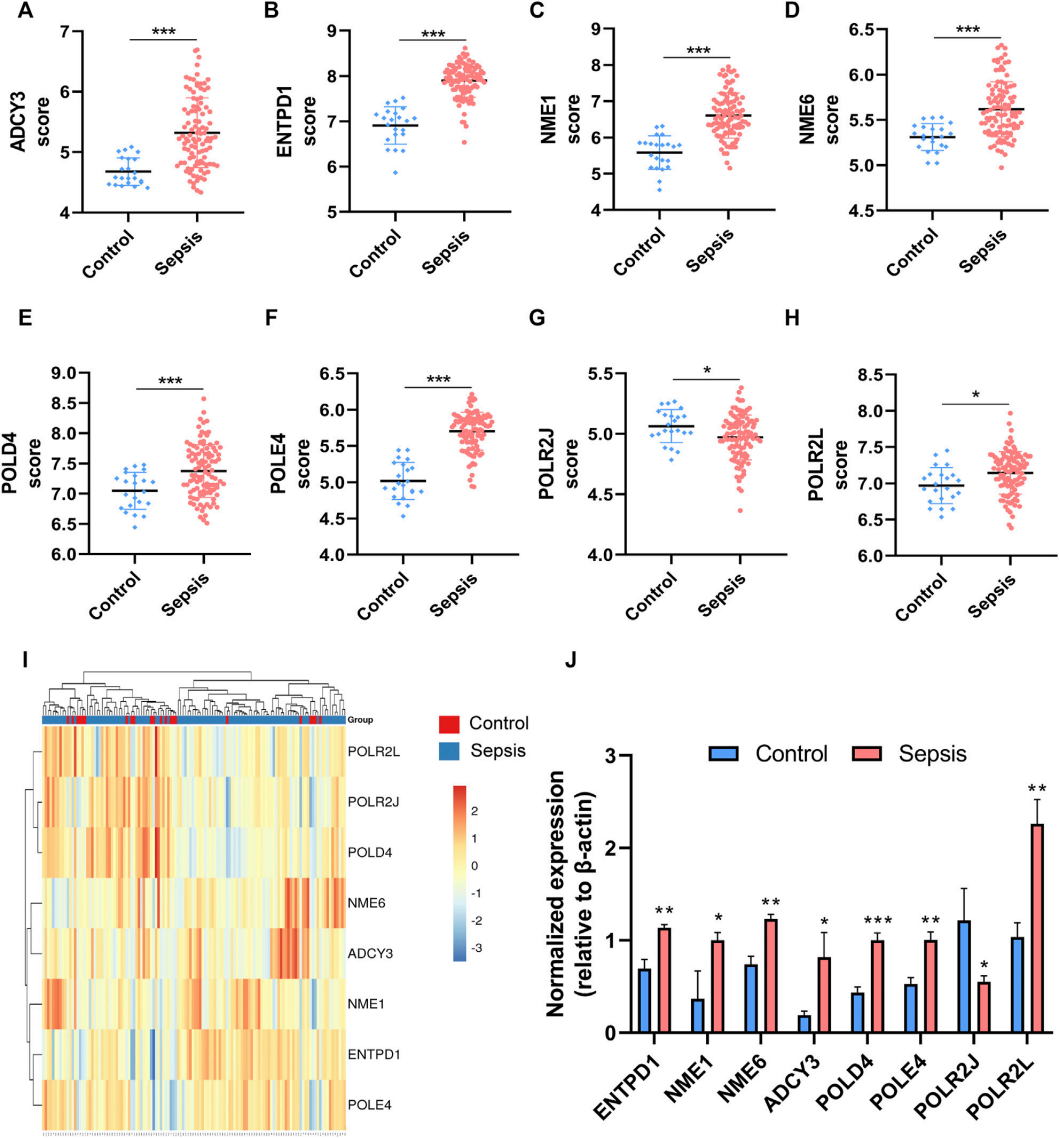
The expressions of the hub genes were different between sepsis patients and healthy controls in the training dataset GSE95233. **(A–H)** Validation of expression of metabolic-related hub genes in patients with sepsis and normal controls in GSE95233. **(I)** Hierarchical clustering analysis demonstrates identified metabolic-related gene expression patterns of heart tissues between septic rat and normal groups in the training cohort. **(J)** Relative expression of identified hub genes in myocardial tissues was compared between septic rats and control rats using quantitative real-time PCR. Differences between two groups were analyzed using the *t*-test. *p < 0.05, **p < 0.01, ***p < 0.001.

To further explore the diagnostic value of these hub genes in sepsis, mRNA levels of eight hub genes were compared in myocardial tissues between septic (*n* = 3) and healthy rats (*n* = 3) with quantitative real-time PCR. The results show that RNA expression levels of seven genes in sepsis were significantly higher than those in health, whereas one gene, *POLR2J*, showed an opposite trend in rats (Figure 5J).

**FIGURE 6:**
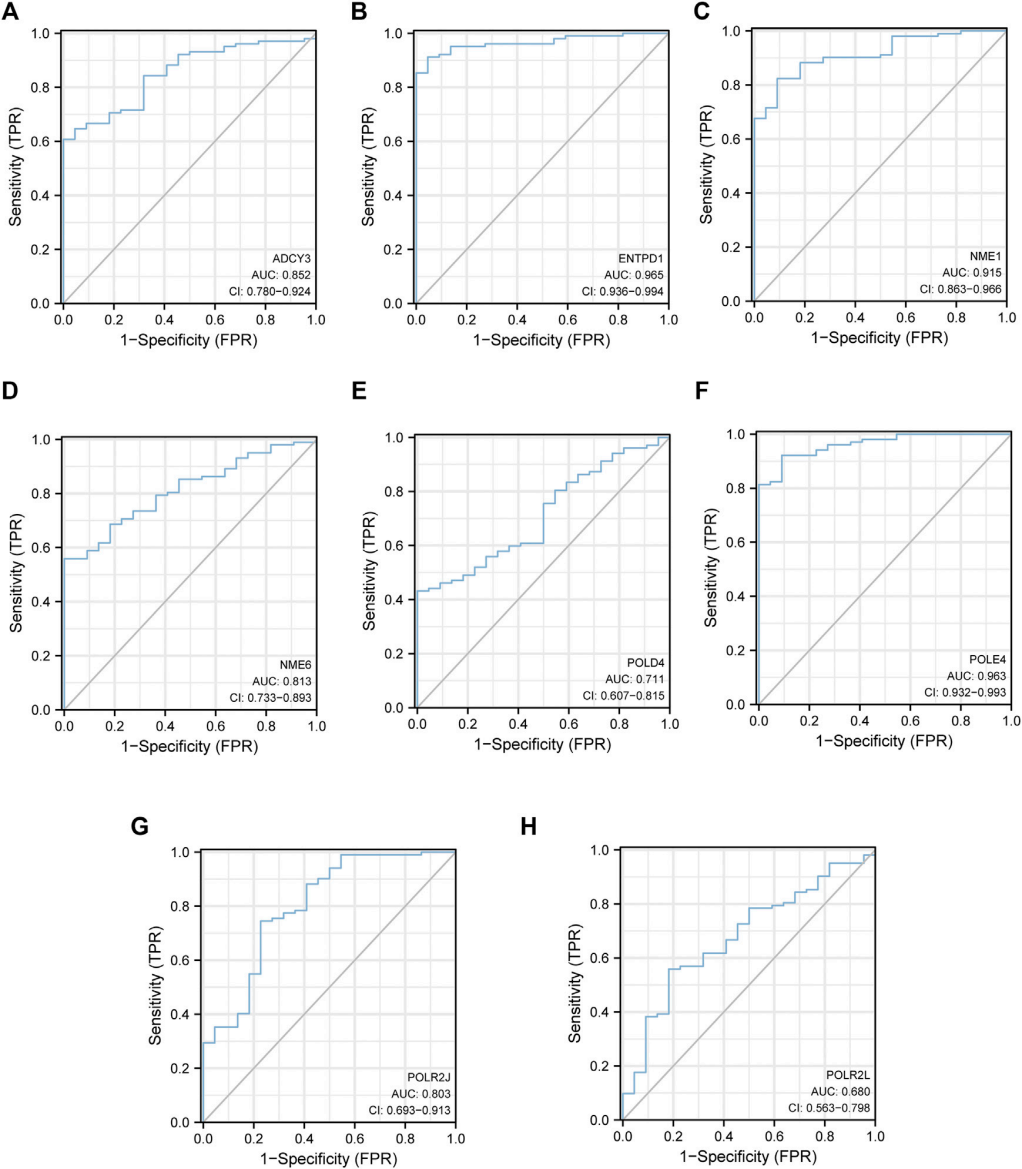
The eight hub genes had potential diagnostic roles in sepsis. **(A)** ROC of *ADCY3*. **(B)** *ENTPD1*. **(C)** *NME1*. **(D)** *NME6*. **(E)** *POLD4*. **(F)** *POLE4*. **(G)** *POLR2J*. **(H)** *POLR2L*.

Hierarchical clustering analysis further revealed that patients with sepsis could be clearly separated into two clusters based on the expression levels of the eight identified hub genes (Figure 5I). Consistent with the trend observed in the hierarchical clustering analysis, the mRNA levels of seven featured genes in sepsis patients were significantly higher than those in the control group except for that of *POLR2J* (Figures 5A–H). To further validate the diagnostic abilities of the identified hub genes for sepsis, ROC curve of SVM classifier was applied to the training dataset. The SVM classifier of the eight hub genes demonstrated excellent discriminatory ability between patients with sepsis and normal controls with high area under the ROC curve, high sensitivity, and specificity (Figure 6). These analyses indicated that the eight hub genes had potential diagnostic roles in sepsis.

### Immune Cell Infiltration Evaluation and Correlation Analysis

**FIGURE 7:**
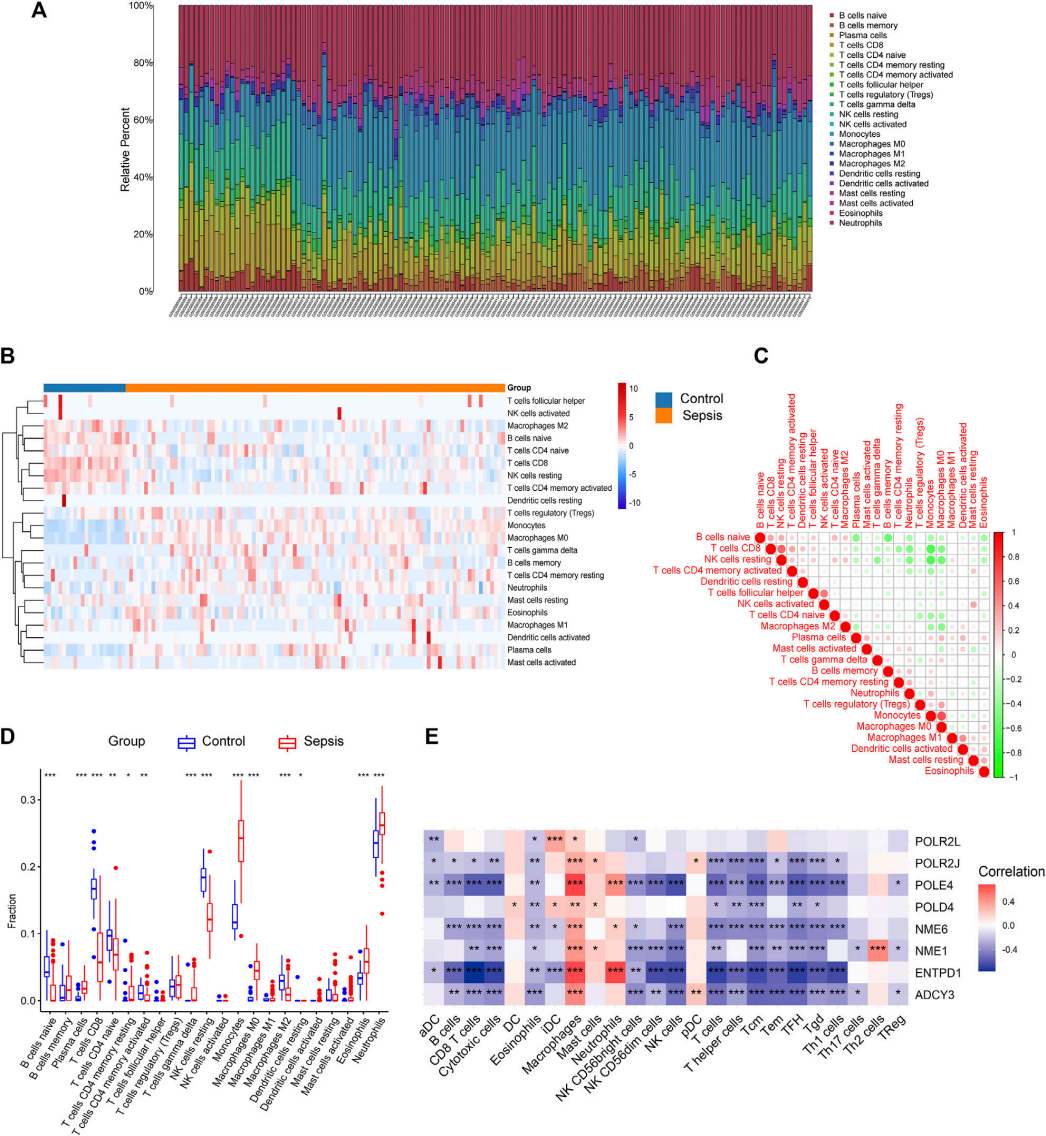
The expressions of the hub genes were correlated with immune cell infiltration in GSE95233. **(A)** Composition and distribution of inferred immune cell infiltration subsets in each sample. **(B)** Heatmap of visualizing the differentially infiltrated immune cells between sepsis and healthy control. The horizontal bar indicated the clustering information of samples that were divided into two major clusters. Vertical bars discriminated between upregulated (red) and downregulated (blue) genes. **(C)** Correlation matrix displaying the Pearson’s correlation values for each comparison between the immune cells. The intensity of the color indicated the strength of the correlation between two immune cells. Red indicates positive correlations and green indicates negative correlations. **(D)** Boxplot of immune-cell proportion comparisons between sepsis patients and healthy controls (the blue and red boxplots stand for control and sepsis, respectively). Abbreviation: CIBERSORT: Cell type identification by estimating relative subsets of RNA transcripts. **(E)** Pearson’s correlation analysis between infiltrating immune cells and identified hub genes. Red nodes indicate positive correlation while blue nodes indicate negative correlation. *p < 0.05, **p < 0.01, ***p < 0.001.

Previous studies demonstrated that immune disorder plays a vital role in the occurrence of sepsis and that metabolic profile determines the immune state (Fitzpatrick and Young, 2013). Therefore, we analyzed the immune cell infiltration proportions, correlation coefficients, and immune-cell proportion comparisons between sepsis patients and healthy controls. We first presented the different immune cell infiltrations by using CIBERSORT in each sample of the training dataset GSE95233 as shown in Figure 7A. Next, the distribution of 22 immune cells between sepsis and healthy control was visualized by a heatmap (Figure 7B). Compared to the healthy control group, the fractions of naïve B cells (*p* < 0.0001), CD8 T cells (*p* < 0.0001), CD4 naïve T cells (*p* < 0.001), CD4 memory-activated T cells (*p* < 0.001), resting NK cells (*p* < 0.0001), M2 macrophages (*p* < 0.0001), and resting dendritic cells (*p* < 0.05) had a lower abundance in patients with sepsis, while the fraction of T cells and plasma cells (*p* < 0.0001), resting CD4 memory (*p* < 0.05), gamma delta T cells (*p* < 0.0001), monocytes (*p* < 0.0001), M0 macrophages (*p* < 0.0001), eosinophils (*p* < 0.0001), and neutrophils (*p* < 0.0001) was significantly higher than those in the control group (Figure 7D). In order to further evaluate whether 22 kinds of immune cells showed convergent effects during infiltration, correlation analysis was used to elucidate the potential one-to-one association. The proportions of different immune cell subpopulations exhibited weak to moderate correlation with each other (Figure 7C). Next, we explored whether the mRNA expression level of identified hub genes was associated with the infiltrations of immune cells in sepsis. The results showed that the expression level of most candidate hub genes was significantly positively correlated with the infiltrating levels of macrophages and significantly negative correlations with B cells, CD8 T cells, cytotoxic cells, eosinophils, NK cells, and most subtypes of T cells. In addition, these hub genes showed weak correlations with DC cells, mast cells, Th17 cells, Th2 cells, and Tregs (Figure 7E). In conclusion, these results revealed the clinical significance of heterogeneous immune cell infiltration in sepsis. Additionally, the association analysis between identified hub genes and immune cell infiltrations indicated an immunosuppressive and exhausted microenvironment in patients with sepsis, which may provide evidence and strategies for immunotherapy.

## Discussion

To date, early cognition of sepsis, prompt completion of the 1-h bundle, and timely administration of broad-spectrum antibiotics have been contributing to the largest reductions in mortality (Evans et al., 2018). Despite significant investments in research and allocation of resources, effective treatments to reduce mortality related to sepsis are still lacking. The challenge in developing strategies to manage sepsis is expected to be related to variability in sepsis presentation due to the complex underlying pathophysiology (Martin-Loeches et al., 2019; Plevin and Callcut, 2017). This is probably one of the reasons why a single biomarker cannot adequately stratify the septic syndrome. Although sepsis is fundamentally defined as an inflammatory disease, it has recently been established that metabolism-related processes can be involved in the pathogenesis of sepsis (Englert and Rogers, 2016). However, the potential role of metabolism-related genes in sepsis remains complex and obscure.

To identify the metabolism-related genes and explore molecular mechanisms contributing to sepsis, we first integrated two gene datasets (GSE95233 and GSE541514) to screen DEGs between sepsis patients and normal controls, and found 66 metabolism-related genes that were differentially expressed in these two datasets (52 upregulated and 14 downregulated DEGs), suggesting that metabolism-related genes may play critical roles in the occurrence of sepsis. GO term analysis indicated that these DEGs were mainly enriched in metabolic processes and pathways. KEGG pathway enrichment analysis revealed alterations in several metabolites of pyrimidine, purine, carbon, sphingolipid, glycerophospholipid, pyruvate, glutathione, and biosynthesis of antibiotics, resulting in the pathogenesis of sepsis. Consistent with functional enrichment analysis of DEGs, these featured DEGs were generally enriched in metabolism-related processes according to the GSEA analysis. Furthermore, a total of 8 hub genes were found in the module with the highest score from the PPI network of all metabolic DEGs, and their functions were mainly associated with nucleoside-diphosphatase activity based on GeneMANIA. Next, these 8 featured metabolism-related genes were identified to allow for good classification distinguished sepsis samples from normal samples using the SVM-RFE algorithm. Therefore, the present study exhibits the potential prognostic value of these featured genes in sepsis, which may provide useful strategies for predicting the outcomes of patients with sepsis.

Previously, inflammatory components of sepsis have been most widely studied and sepsis has been considered primarily as dysregulated immune response to infection. *Matrix metallopeptidase 9* (*MMP9*) and *Complement C3a Receptor 1* (*C3AR1*), immune infiltration-associated biomarkers of sepsis, have been reported to be related with the prognosis of sepsis patients (Xu et al., 2020). However, recent studies have shown that the pathogenesis of sepsis is clearly influenced by profound changes in metabolic homeostasis (Van Wyngene et al., 2018a). Therefore, novel sepsis biomarkers associated with metabolism need to be explored. In our study, the metabolomics network of the compound, gene, enzyme, and reaction regulated by the hub genes was first conducted, and it indicated that the featured hub genes play a pivotal role in the interplays among the dysregulated metabolites in sepsis. We further found that the metabolic pathways of hub genes were essentially involved in purine metabolism, pyrimidine metabolism, GAP-filling DNA repair synthesis and ligation in TC-NER, nucleobase-containing small-molecule biosynthetic process, and HTLV-I infection, which was further validated by the LC-MS-based untargeted metabolomics analysis between septic rat myocardial tissues and normal myocardial tissues.

*NME1* plays a role in lipid metabolism. *NME1* has been found to bind to porcine ST8 alpha-N-acetyl-neuraminide alpha-2,8-sialyltransferase 1 (pST8SIA1), inhibiting ganglioside GD3 synthesis and neuronal differentiation (Cho et al., 2021). As lipid metabolism is impaired in sepsis (Chung et al., 2017), *NME1* may be involved in the pathophysiology of sepsis, in accordance with our finding showing that *NME1* expression is elevated in sepsis patients compared with healthy controls. *NME6*, another member of NME family, is associated with ATP metabolism (Rewcastle et al., 1996). ATP release occurs during sepsis and activates macrophages, exacerbating prognosis of sepsis (Dosch et al., 2019). Therefore, *NME6* may influence the outcome of sepsis by involving ATP metabolism.

*POLD4* encodes the smallest subunit of DNA polymerase delta and is a metabolism-related gene (Liang et al., 2018). The genomic instability caused by *POLD4* may exacerbate the prognosis of sepsis patients. *POLE4* is a histone H3–H4 chaperone that maintains chromatin integrity during DNA replication (Bellelli et al., 2018), and may be involved in DNA metabolism (Abdelmohsen et al., 2012). However, the exact role of *POLE4* in the pathophysiology of sepsis remains unclear.

*POLR2J* and *POLR2L* encode two of the subunits of RNA polymerase II, which is responsible for synthesizing messenger RNA in eukaryotes. *POLR2J* and *POLR2L* are associated with purine metabolism and pyrimidine metabolism in sepsis patients (Zhang et al., 2017). In the present study, we also identified *POLR2J* and *POLR2L* as two of the hub genes of sepsis, indicating the important role of these two metabolism-related genes in the pathophysiology of sepsis.

*Adenylate cyclase 3 (ADCY3)* is a membrane-associated enzyme and catalyzes the formation of the secondary messenger cyclic adenosine monophosphate (cAMP), being involved in pathophysiological metabolic processes. *ADCY3* has been found to be increased in human dendritic cells in the condition of inflammation (Midttun et al., 2018). However, the exact role of *ADCY3* in the metabolic changes in sepsis has not been identified.

*Ectonucleoside Triphosphate diphosphohydrolase 1 (ENTPD1)* hydrolyzes extracellular ATP and ADP to AMP and is related to ATP metabolism and purine metabolism (Bastid et al., 2013). We identified *ENTPD1* as one of the hub genes of sepsis, in accordance with a study that shows that *ENTPD1* increases extracellular ATP metabolism, inhibits inflammatory signaling, and attenuates sepsis-associated liver injury (Savio et al., 2017).

These metabolic-related genes may become potential biomarkers of sepsis. Whether these genes participate in the pathophysiology of sepsis needs to be further studied. Targeting these genes may improve outcomes of sepsis patients.

As immune cells can switch back and forth between different metabolic states and preferentially utilize specific metabolites to sustain the relative functions of their effectors (Ganeshan and Chawla, 2014), we then determined the inferred immune cell infiltration proportions, correlation coefficients, and immune-cell proportion comparisons between sepsis patients and healthy controls. Compared with the control group, the fractions of naive B cells, CD8 T cells, naive CD4 T cells, memory-activated CD4 T cells, resting NK cells, M2 macrophages, and resting dendritic cells had a lower abundance in patients with sepsis, while the fractions of T cells, plasma cells, resting CD4 memory, gamma delta T cells, monocytes, M0 macrophages, eosinophils, and neutrophils were significantly higher than those in the control group, indicating that acute inflammation and cell immunity play a crucial role in the pathophysiology of sepsis. Reprogramming of metabolism has been shown to be associated with the changes of immune cell infiltration proportions (Van Wyngene et al., 2018b). However, the exact metabolic pathways through which the 8 hub genes affect immune cell infiltration in sepsis patients need to be further studied. All in all, the clinical significance of heterogeneous immune cell infiltration will expand our understanding of the molecular mechanism underlying the occurrence of sepsis and may provide novel evidence and strategies for immunotherapy.

In conclusion, using a bioinformatics analysis of two transcriptomic datasets (GSE95233 and GSE54514), we identified the immune characteristics and the metabolic mechanism of sepsis. The heterogeneities of immune cell infiltration could provide new insights into sepsis immunotherapy. Meanwhile, the present study identified eight metabolism-related genes (*NME1, NME6, POLD4, POLE4, POLR2J, POLR2L, ADCY3*, and *ENTPD1*) in patients with sepsis and the identified metabolism-related genes may represent diagnostic and therapeutic biomarkers of sepsis. Knowledge of these genes will improve our understanding of the molecular mechanism underlying the occurrence of sepsis.

## Data Availability

The datasets presented in this study can be found in online repositories. The names of the repository/repositories and accession number(s) can be found at: https://www.ncbi.nlm.nih.gov/, GSE95233, and https://www.ncbi.nlm.nih.gov/, GSE54514.

## References

[B1] Abdelmohsen K. Srikantan S. Tominaga K. Kang M.-J. Yaniv Y. Martindale J. L. (2012). Growth Inhibition by miR-519 via Multiple P21-Inducing Pathways. Mol. Cel Biol 32 (13), 2530–2548. 10.1128/mcb.00510-12 PMC3434494 22547681

[B2] Bastid J. Cottalorda-Regairaz A. Alberici G. Bonnefoy N. Eliaou J.-F. Bensussan A. (2013). ENTPD1/CD39 Is a Promising Therapeutic Target in Oncology. Oncogene 32 (14), 1743–1751. 10.1038/onc.2012.269 22751118

[B3] Bellelli R. Belan O. Pye V. E. Clement C. Maslen S. L. Skehel J. M. (2018). POLE3-POLE4 Is a Histone H3-H4 Chaperone that Maintains Chromatin Integrity during DNA Replication. Mol. Cel 72 (1), 112–e5. 10.1016/j.molcel.2018.08.043 PMC6179962 30217558

[B4] Chen C. Chen H. Zhang Y. Thomas H. R. Frank M. H. He Y. (2020). TBtools: An Integrative Toolkit Developed for Interactive Analyses of Big Biological Data. Mol. Plant 13 (8), 1194–1202. 10.1016/j.molp.2020.06.009 32585190

[B5] Cho J. H. Ju W. S. Seo S. Y. Kim B. H. Kim J.-S. Kim J.-G. (2021). The Potential Role of Human NME1 in Neuronal Differentiation of Porcine Mesenchymal Stem Cells: Application of NB-hNME1 as a Human NME1 Suppressor. Ijms 22 (22), 12194. 10.3390/ijms222212194 34830075 PMC8619003

[B6] Chung K. W. Kim K. M. Choi Y. J. An H. J. Lee B. Kim D. H. (2017). The Critical Role Played by Endotoxin-Induced Liver Autophagy in the Maintenance of Lipid Metabolism during Sepsis. Autophagy 13 (7), 1113–1129. 10.1080/15548627.2017.1319040 28575583 PMC5529074

[B7] Débora Maria da Gomes Cunha G. Gd. S. Yoshio Hamasaki Mike. (2019). New Biomarkers of Sepsis with Clinical Relevance, Clinical Management of Shock - the Science and Art of Physiological Restoration. 10.5772/intechopen.82156

[B8] Djande C. Y. H. Pretorius C. Tugizimana F. Piater L. A. Dubery I. A (2020). Metabolomics: A Tool for Cultivar Phenotyping and Investigation of Grain Crops. Agronomy 10 (6), 831. 10.3390/agronomy10060831

[B9] Dosch M. Zindel J. Jebbawi F. Melin N. Sanchez-Taltavull D. Stroka D. (2019). Connexin-43-dependent ATP Release Mediates Macrophage Activation during Sepsis. Elife 88. 10.7554/eLife.42670 PMC6415938 30735126

[B10] Englert J. A. Rogers A. J. (2016). Metabolism, Metabolomics, and Nutritional Support of Patients with Sepsis. Clin. Chest Med. 37 (2), 321–331. 10.1016/j.ccm.2016.01.011 27229648 PMC5084839

[B11] Evans I. V. R. Phillips G. S. Alpern E. R. Angus D. C. Friedrich M. E. Kissoon N. (2018). Association between the New York Sepsis Care Mandate and In-Hospital Mortality for Pediatric Sepsis. Jama 320 (4), 358–367. 10.1001/jama.2018.9071 30043064 PMC6500448

[B12] Fitzpatrick M. Young S. P. (2013). Metabolomics--a Novel Window into Inflammatory Disease. Swiss Med. Wkly 143, w13743. 10.4414/smw.2013.13743 23348753 PMC4337982

[B13] Fleischmann C. Scherag A. Adhikari N. K. Hartog C. S. Tsaganos T. Schlattmann P. (2016). Assessment of Global Incidence and Mortality of Hospital-Treated Sepsis. Current Estimates and Limitations. Am. J. Respir. Crit. Care Med. 193 (3), 259–272. 10.1164/rccm.201504-0781OC 26414292

[B14] Ganeshan K. Chawla A. (2014). Metabolic Regulation of Immune Responses. Annu. Rev. Immunol. 32, 609–634. 10.1146/annurev-immunol-032713-120236 24655299 PMC5800786

[B15] Hamasaki M. Y. Barbeiro H. V. de Souza H. P. Machado M. C. da Silva F. P. (2014). sRAGE in Septic Shock: a Potential Biomarker of Mortality. Rev. Bras Ter Intensiva 26 (4), 392–396. 10.5935/0103-507X.20140060 25607269 PMC4304468

[B16] Itenov T. S. Murray D. D. Jensen J. U. S. (2018). Sepsis: Personalized Medicine Utilizing 'Omic' Technologies-A Paradigm Shift? Healthcare (Basel) 6 (3). 10.3390/healthcare6030111 PMC6163606 30205441

[B17] Jacob M. Lopata A. L. Dasouki M. Abdel Rahman A. M. (2019). Metabolomics toward Personalized Medicine. Mass. Spec. Rev. 38 (3), 221–238. 10.1002/mas.21548 29073341

[B18] Koutroulis I. Batabyal R. McNamara B. Ledda M. Hoptay C. Freishtat R. J. (2019). Sepsis Immunometabolism: From Defining Sepsis to Understanding How Energy Production Affects Immune Response. Crit. Care Explor 1 (11), e0061. 10.1097/CCE.0000000000000061 32166242 PMC7063962

[B19] Lewis A. J. Billiar T. R. Rosengart M. R. (2016). Biology and Metabolism of Sepsis: Innate Immunity, Bioenergetics, and Autophagy. Surg. infections 17 (3), 286–293. 10.1089/sur.2015.262 PMC4876546 27093228

[B20] Liang J. Q. Teoh N. Xu L. Pok S. Li X. Chu E. S. H. (2018). Dietary Cholesterol Promotes Steatohepatitis Related Hepatocellular Carcinoma through Dysregulated Metabolism and Calcium Signaling. Nat. Commun. 9 (1), 4490. 10.1038/s41467-018-06931-6 30367044 PMC6203711

[B21] Martin-Loeches I. Guia M. C. Vallecoccia M. S. Suarez D. Ibarz M. Irazabal M. (2019/02/04 2019). Risk Factors for Mortality in Elderly and Very Elderly Critically Ill Patients with Sepsis: a Prospective, Observational, Multicenter Cohort Study. Ann. Intensive Care 9 (1), 26. 10.1186/s13613-019-0495-x PMC6362175 30715638

[B22] Metsalu T. Vilo J. (2015). ClustVis: a Web Tool for Visualizing Clustering of Multivariate Data Using Principal Component Analysis and Heatmap. Nucleic Acids Res. 43 (W1), W566–W570. 10.1093/nar/gkv468 25969447 PMC4489295

[B23] Michie H. R. (1996). Metabolism of Sepsis and Multiple Organ Failure. World J. Surg. 20 (4), 460–464. 10.1007/s002689900072 8662135

[B24] Midttun H. L. E. Ramsay A. Mueller-Harvey I. Williams A. R. (2018). Cocoa Procyanidins Modulate Transcriptional Pathways Linked to Inflammation and Metabolism in Human Dendritic Cells. Food Funct. 9 (5), 2883–2890. 10.1039/c8fo00387d 29714395

[B25] Nedeva C. Menassa J. Puthalakath H. (2019). Sepsis: Inflammation Is a Necessary Evil. Front. Cel Dev. Biol. 7, 108. 10.3389/fcell.2019.00108 PMC6596337 31281814

[B26] Oberhoffer M. Vogelsang H. Russwurm S. Hartung T. Reinhart K. (1999). Outcome Prediction by Traditional and New Markers of Inflammation in Patients with Sepsis. Clin. Chem. Lab. Med. 37 (3), 363–368. 10.1515/CCLM.1999.060 10353484

[B27] Parnell G. P. Tang B. M. Nalos M. Armstrong N. J. Huang S. J. Booth D. R. (2013). Identifying Key Regulatory Genes in the Whole Blood of Septic Patients to Monitor Underlying Immune Dysfunctions. Sep 40 (3), 166–174. 10.1097/shk.0b013e31829ee604 23807251

[B28] Phua J. Koay E. S. C. Lee K. H. (2008). Lactate, Procalcitonin, and Amino-Terminal Pro-b-type Natriuretic Peptide versus Cytokine Measurements and Clinical Severity Scores for Prognostication in Septic Shock. Mar 29 (3), 328–333. 10.1097/shk.0b013e318150716b 18277855

[B29] Pinheiro da Silva F. César Machado M. C. (2015). Personalized Medicine for Sepsis. Am. J. Med. Sci. 350 (5), 409–413. 10.1097/maj.0000000000000558 26398478

[B30] Plevin R. Callcut R. (2017). Update in Sepsis Guidelines: what Is Really New? Trauma Surg. Acute Care Open 2 (1), e000088. 10.1136/tsaco-2017-000088 29766091 PMC5877904

[B31] Pop-Began V. Păunescu V. Grigorean V. Pop-Began D. Popescu C. (2014). Molecular Mechanisms in the Pathogenesis of Sepsis. J. Med. Life 7, 38–41. 10.4172/2155-9899.S1.019 25870671 PMC4391358

[B32] Rewcastle G. W. Palmer B. D. Thompson A. M. Bridges A. J. Cody D. R. Zhou H. (1996). Tyrosine Kinase Inhibitors. 10. Isomeric 4-[(3-Bromophenyl)amino]pyrido[d]-Pyrimidines Are Potent ATP Binding Site Inhibitors of the Tyrosine Kinase Function of the Epidermal Growth Factor Receptor. J. Med. Chem. 39 (9), 1823–1835. 10.1021/jm9508651 8627606

[B33] Rhodes A. Evans L. E. Alhazzani W. Levy M. M. Antonelli M. Ferrer R. (20162017). Surviving Sepsis Campaign: International Guidelines for Management of Sepsis and Septic Shock: 2016Critical Care Medicine. Crit. Care Med. 45 (3), 486–552. 10.1097/CCM.0000000000002255 28098591

[B34] Rudd K. E. Kissoon N. Limmathurotsakul D. Bory S. Mutahunga B. Seymour C. W. (2018). The Global burden of Sepsis: Barriers and Potential Solutions. Crit. Care 22 (1), 232. 10.1186/s13054-018-2157-z 30243300 PMC6151187

[B35] Savio L. E. B. de Andrade Mello P. Figliuolo V. R. de Avelar Almeida T. F. Santana P. T. Oliveira S. D. S. (2017). CD39 Limits P2X7 Receptor Inflammatory Signaling and Attenuates Sepsis-Induced Liver Injury. J. Hepatol. 67 (4), 716–726. 10.1016/j.jhep.2017.05.021 28554875 PMC5875702

[B36] Scott M. C. (2017). Defining and Diagnosing Sepsis. Emerg. Med. Clin. North America 35 (1), 1–9. 10.1016/j.emc.2016.08.002 27908326

[B37] Singer M. De Santis V. Vitale D. Jeffcoate W. (2004). Multiorgan Failure Is an Adaptive, Endocrine-Mediated, Metabolic Response to Overwhelming Systemic Inflammation. Lancet 364 (9433), 545–548. 10.1016/S0140-6736(04)16815-3 15302200

[B38] Singer M. (2014). The Role of Mitochondrial Dysfunction in Sepsis-Induced Multi-Organ Failure. Virulence 5 (1), 66–72. 10.4161/viru.26907 24185508 PMC3916385

[B39] Singer M. Deutschman C. S. Seymour C. W. Shankar-Hari M. Annane D. Bauer M. (2016). The Third International Consensus Definitions for Sepsis and Septic Shock (Sepsis-3). Jama 315 (8), 801–810. 10.1001/jama.2016.0287 26903338 PMC4968574

[B40] Tabone O. Mommert M. Jourdan C. Cerrato E. Legrand M. Lepape A. (2018). Endogenous Retroviruses Transcriptional Modulation after Severe Infection, Trauma and Burn. Front. Immunol. 9, 3091. 10.3389/fimmu.2018.03091 30671061 PMC6331457

[B41] Van Wyngene L. Vandewalle J. Libert C. (2018). Reprogramming of Basic Metabolic Pathways in Microbial Sepsis: Therapeutic Targets at Last? EMBO Mol. Med. 10 (8), e8712. 10.15252/emmm.201708712 29976786 PMC6079534

[B42] Van Wyngene L. Vandewalle J. Libert C. (2018). Reprogramming of Basic Metabolic Pathways in Microbial Sepsis: Therapeutic Targets at Last? EMBO Mol. Med. 10 (8). 10.15252/emmm.201708712 PMC6079534 29976786

[B43] Venet F. Monneret G. (2018). Advances in the Understanding and Treatment of Sepsis-Induced Immunosuppression. Nat. Rev. Nephrol. 14 (2), 121–137. 10.1038/nrneph.2017.165 29225343

[B44] Xie J. Wang H. Kang Y. Zhou L. Liu Z. Qin B. (2020). The Epidemiology of Sepsis in Chinese ICUs. Crit. Care Med. 48 (3), e209–e218. 10.1097/ccm.0000000000004155 31804299

[B45] Xu C. Xu J. Lu L. Tian W. Ma J. Wu M. (2020). Identification of Key Genes and Novel Immune Infiltration-Associated Biomarkers of Sepsis. Innate Immun. 26 (8), 666–682. 10.1177/1753425920966380 33100122 PMC7787554

[B46] Zhang A. Sun H. Yan G. Wang P. Wang X. (2015). Metabolomics for Biomarker Discovery: Moving to the Clinic. Biomed. Res. Int. 2015, 354671. 10.1155/2015/354671 26090402 PMC4452245

[B47] Zhang J. Cheng Y. Duan M. Qi N. Liu J. (2017). Unveiling Differentially Expressed Genes upon Regulation of Transcription Factors in Sepsis. 3 Biotech. 7 (1), 46. 10.1007/s13205-017-0713-x PMC5428098 28444588

[B48] Zhu Y. Wu H. Wu Y. Zhang J. Peng X. Zang J. (2016). Beneficial Effect of Intermedin 1-53 in Septic Shock Rats. Shock. 46 (5), 557–565. 10.1097/shk.0000000000000639 27355401

