# Supplementary figures and images for: The Landscape of Featured Metabolism-Related Genes and Imbalanced Immune Cell Subsets in Sepsis
Source: Front Genet. 2022 Feb 21;13:821275. doi: 10.3389/fgene.2022.821275 (PMC8901109; doi:10.3389/fgene.2022.821275)

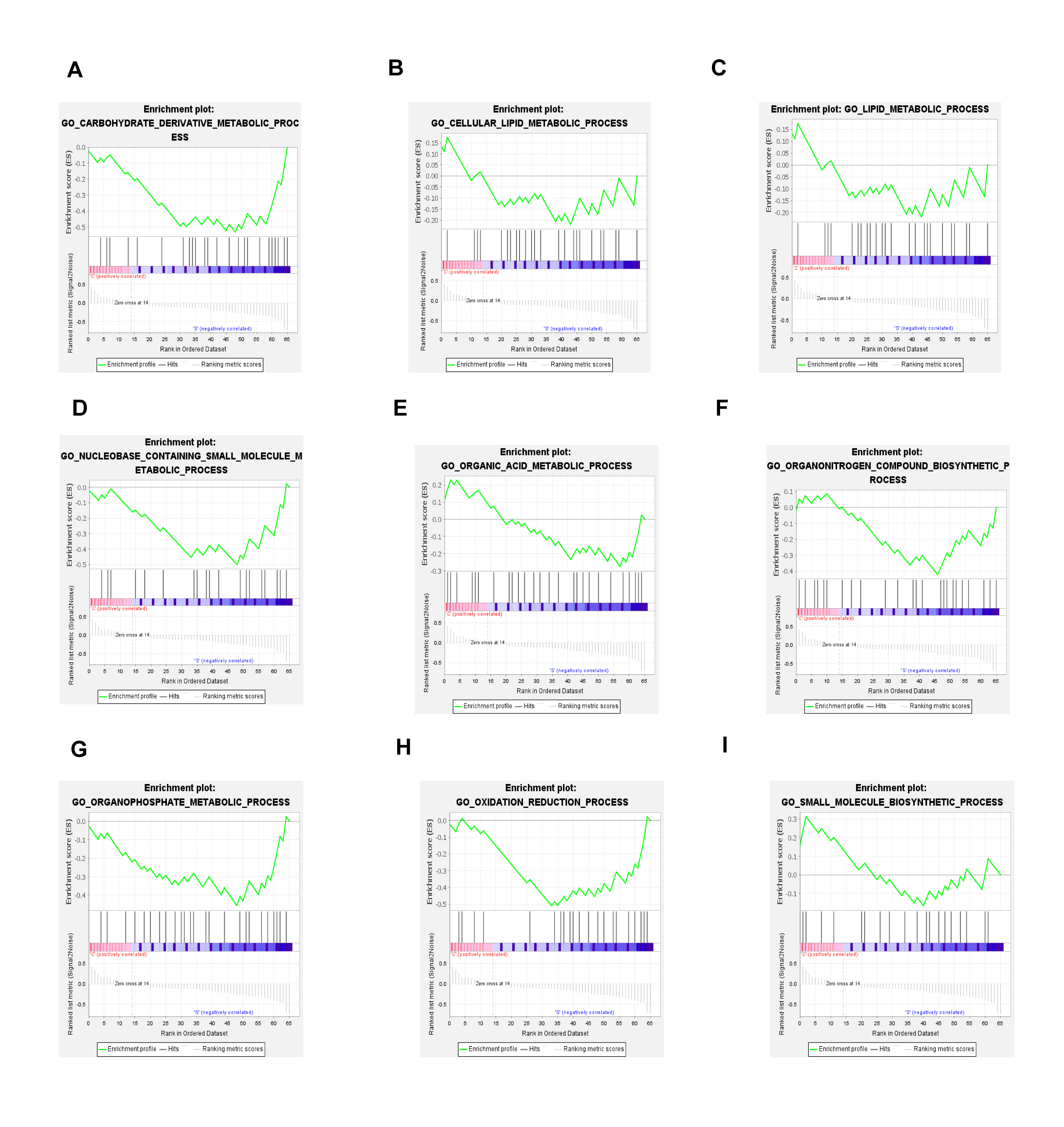

Supplement: Supplementary file 1 [file Image2.TIF]

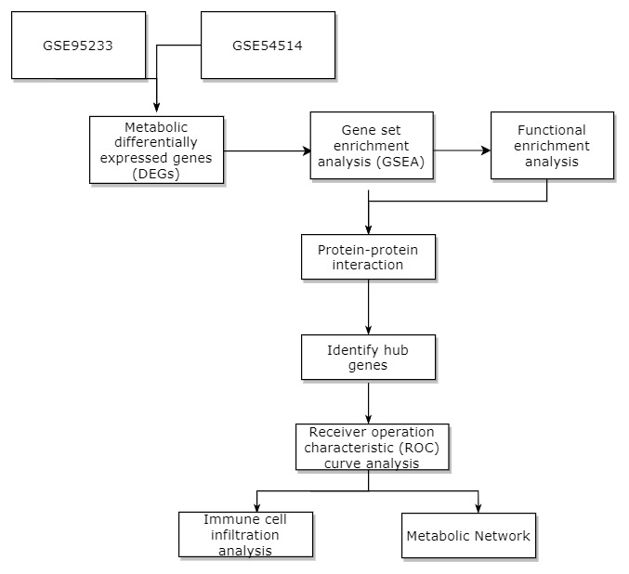

Supplement: Supplementary file 2 [file Image1.TIF]
